# Supplementary material for: Deletion of wfs1 Impairs Oligodendrocyte Precursor Cells Dorsal Distribution and Myelination Through the wfs1-hmgcs1 Axis in Zebrafish
Source: Biology (Basel). 2026 Aug 16;15(16):1402. doi: 10.3390/biology15161402 (PMC13510151; doi:10.3390/biology15161402)
Supplement: Supplementary file 1 [file biology-15-01402-s001.zip › File S1-Transcriptome sequencing data and differentially expressed genes (DEGs) in wfs1-- zebrafish at 5 dpf/1.GO/wfs1KOvsWT/UP/wfs1KOvsWT.up.GOmf_DAG.pdf]

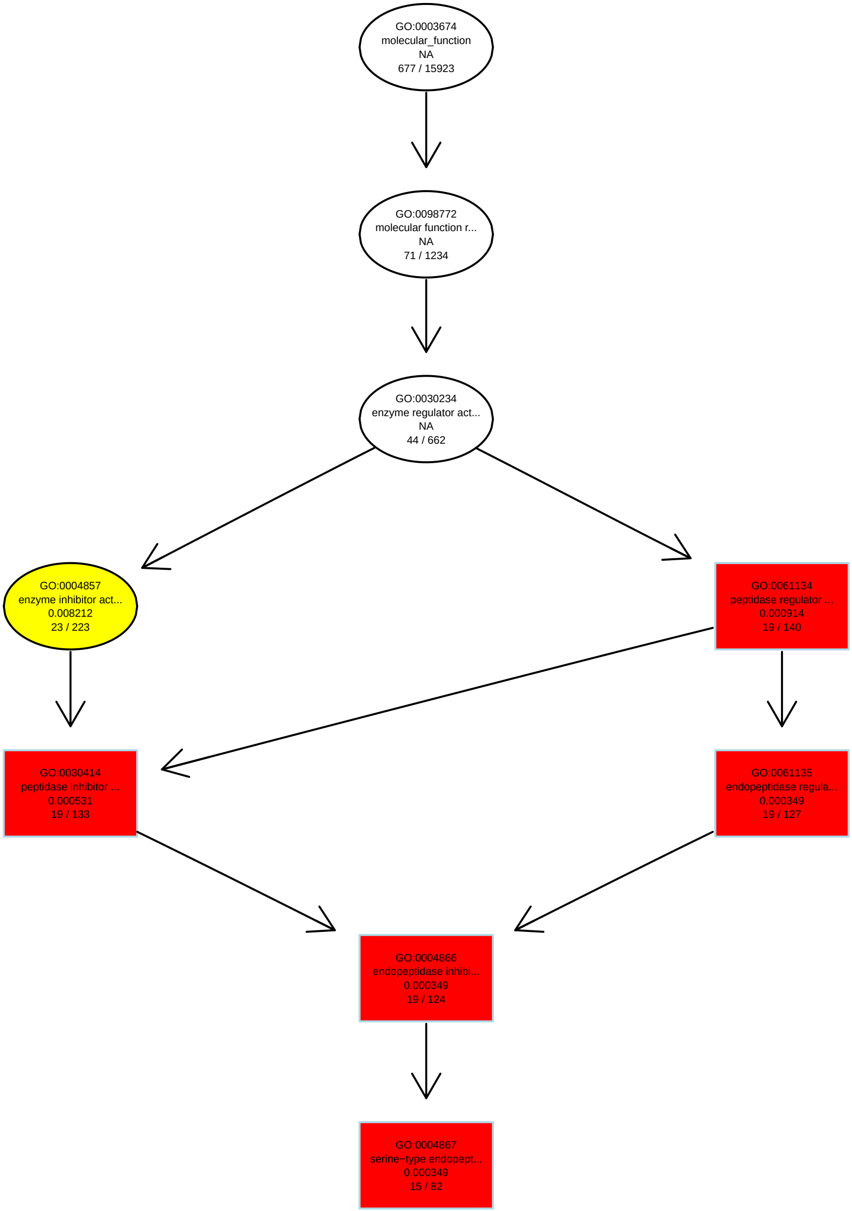
GO:0003674
molecular_function
NA
677 / 15923
GO:0098772
molecular function r...
NA
71 / 1234
GO:0030234
enzyme regulator act...
NA
44 / 662
GO:0004857
enzyme inhibitor act...
0.008212
23 / 223
GO:0061134
peptidase regulator ...
0.000914
19 / 140
GO:0030414
peptidase inhibitor ...
0.000531
19 / 133
GO:0061135
endopeptidase regula...
0.000349
19 / 127
GO:0004866
endopeptidase inhibi...
0.000349
19 / 124
GO:0004867
serine−type endopept...
0.000349
15 / 82
